# MicroRNA-related sequence variations in human cancers

**DOI:** 10.1007/s00439-013-1397-x

**Published:** 2013-11-19

**Authors:** A. Wojcicka, A. de la Chapelle, K. Jazdzewski

**Affiliations:** 1 Genomic Medicine, Department of General, Transplant, and Liver Surgery, Medical University of Warsaw, Zwirki i Wigury 61, 02-091 Warsaw, Poland; 2 Laboratory of Human Cancer Genetics, Centre of New Technologies, CENT, University of Warsaw, 02-089 Warsaw, Poland; 3 Human Cancer Genetics Program, Comprehensive Cancer Center, Ohio State University, Columbus, OH 43210 USA

## Abstract

MicroRNAs are emerging as a most promising field in basic and translational research, explaining the pathogenesis of numerous human diseases and providing excellent tools for their management. This review considers the effects of microRNA sequence variations and their implication in pathogenesis and predisposition to human cancers. Although the role of microRNAs still remains to be elucidated, functional, and populational studies indicate that microRNA variants are important factors underlying the process of carcinogenesis. Further understanding of the cellular and molecular basis of microRNA action will lead to the identification of their new target genes and microRNA-regulated pathways. As a consequence, novel models of cancer pathogenesis can be proposed, and serve as a basis for elucidation of new prognostic and diagnostic tools for human cancers.

## MicroRNA action

The discovery of microRNAs (miRNAs, miRs) was a milestone in our quest to understand the pathogenesis of numerous human cancers. Deregulation of gene expression observed in malignancies can nowadays be explained by the action of short, endogenous non-coding RNAs–microRNAs. The cellular “microcosm” is emerging as a most promising field in translational research, providing excellent diagnostic, prognostic and therapeutic tools for the management of human diseases.

MicroRNAs are encoded all over the human genome—in intergenic regions as well as within introns or exons of protein-coding genes (Rodriguez et al. 2004). Primary miRNA transcripts are processed to produce miRNA precursors (pre-miRNAs) that fold into specific secondary structures of hairpins. Pre-miRNAs are further cleaved, and mature microRNAs are produced from one or both arms of the hairpin. The produced miRs are referred to as miR-5p and miR-3p, depending on the arm from which they are cleaved (Bartel 2009). In animals, miRNAs act mainly through annealing to 3′ untranslated regions (3′UTRs) of gene transcripts, leading to inhibition of further steps of gene expression. This interaction depends on Watson–Crick complementarity between the target 3′UTR sequence and the “seed” region, located between the second and eighth nucleotide of the mature miRNA (Friedman et al. 2009; Grimson et al. 2007). It is believed that microRNAs regulate the expression of approximately 50 % of human genes (Krol et al. 2010).

To date, there are 1,872 precursors and 2,578 mature miRNAs annotated and described in humans (miRbase release 20) (Griffiths-Jones et al. 2008; Kozomara and Griffiths-Jones 2011). According to a recent analysis, the pre-microRNA regions include 1,940 SNPs. Moreover, 414,510 other human SNPs might potentially influence miR:mRNA interactions, causing either loss or creation of a miRNA-binding site (Liu et al. 2012). Considering the prevalence of microRNA-mediated gene regulation, sequence variations in miRs can significantly contribute to changes in important cellular pathways and thus underlie diseases. Genetic variations in microRNA sequences are unique as they can influence both the expression levels and functionality of a miRNA. A SNP located in the crucial “seed” sequence of a miR affects its complementarity with target genes and leads to deregulation of multiple cellular pathways. Moreover, since the expression of miRs is highly tissue and disease-specific, changes within the miRNA sequence can indeed specifically predispose to cancers of particular organs and mediate different molecular changes in different tissues (de la Chapelle and Jazdzewski 2011).

The first microRNAs whose aberrations were linked to the pathogenesis of cancer were miR-15a and miR-16-1, lost in the majority of B cell chronic lymphocytic leukemia (CLL) patients due to the deletion of chromosome 13q14 (Calin et al. 2002). Later studies revealed an alternative mechanism leading to severe downregulation of the miRs in a few cases of CLL, namely, a germline C → T substitution downstream of the miR-16-1 hairpin (Calin et al. 2005). The explanation of the downregulation was unknown till recently, when Auyeung et al. (2013) showed that specific sequence determinants, including a recognition site for the splicing factor SRp20 located downstream of most pri-miRNA hairpins, are required for efficient processing and maturation of microRNAs in human cells. The deleterious mutation lowers the expression of miR-16-1 and leads to CLL through the disruption of SRp20 binding site.

## SNPs in microRNA genes

The first reports on variations in microRNA sequences were published more than 10 years after the discovery of microRNAs and were first proposed to have no effect on the functionality of miRs (Iwai and Naraba 2005; Saunders et al. 2007). Naturally occurring sequence variation in a miRNA precursor that resulted in reduced processing, lowered levels, and disrupted function of a mature miRNA was first reported for a viral miR-K5 (Gottwein et al. 2006) and, subsequently, for human miR-125a (Duan et al. 2007) and miR-146a (Jazdzewski et al. 2008).

All these studies proved that a single nucleotide polymorphism that leads to disruption of base pairing in the hairpin stem, results in a dramatically impaired processing and downregulation of the mature microRNA. The rs12975333 in miR-125a results in a G → T change at the eighth nucleotide of the mature miR and severely reduces its production (Duan et al. 2007). The polymorphism was shown to be associated with a significantly increased risk for breast cancer in Antwerp (Li et al. 2009). It was proposed that lowered levels of the mature miR-125a lead to overexpression of its target gene, HER2, whose increased levels are implicated in numerous breast cancer cases (Lehmann et al. 2013). Interestingly, a large multi-center study performed in Germany, Italy, Austria, and Spain did not identify any carriers of the rare rs12975333 allele to confirm these results (Peterlongo et al. 2011).

The rs2910164 G → C variant in miR-146a is a single nucleotide polymorphism whose deleterious function is mediated by two mechanisms: by impaired processing and downregulation of mature microRNA levels resulting in a diminished regulatory impact on target genes (Jazdzewski et al. 2008), and by creation of a new variant of the mature microRNA (Jazdzewski et al. 2009). Mature miRs are produced from both the 5p and 3p arms of the precursor mir-146a. The SNP is located in the seed region of miR-146a-3p, generating 2 isoforms that regulate distinct sets of target genes. Thus, homozygous carriers of GG or CC alleles produce 2 mature molecules (miR-146a-5p from the leading strand, and miR-146a-3p(G) or 146a-3p(C), respectively, from the passenger strand), whereas GC heterozygotes produce 3 mature miRs: miR-146a-5p and both miR-146a-3p(G) and miR-146a-3p(C).

Importantly, 4.7 % of thyroid tumors exhibited a somatic mutation in the SNP sequence that probably arose as a step in the clonal selection during carcinogenesis (Jazdzewski et al. 2008). Strikingly, all the identified somatic mutations occurred toward heterozygosity. The authors suggested that thyroid cells heterozygous at miR-146a presented higher activity of NF-kappaB and a lowered potential of inhibition of this pathway; therefore were more likely to survive after ionizing radiation, a known risk factor for thyroid cancer (Jazdzewski and de la Chapelle 2009).

The rs2910164 in miR-146a was shown to significantly predispose to papillary thyroid carcinoma (Jazdzewski et al. 2008), hepatocellular carcinoma (HCC) in males (Xu et al. 2008), prostate cancer (Xu et al. 2010), bladder cancer (Wang et al. 2012), and colorectal cancer (Ma et al. 2013), although not in all studied populations (Jones et al. 2012). It was also suggested that rs2910164 can significantly contribute to the pathogenesis of breast cancer, as the target genes of miR-146a include *BRCA1* and *BRCA2*, which are key breast and ovarian cancer genes. Breast cancer patients who had at least one miR-146a-variant allele were diagnosed at an earlier age than the patients with no variant alleles (Shen et al. 2008).

To what extent sequence variations in microRNA genes are associated with tumorigenesis became a matter of intensive investigation. Rs11614913 (C → T) in a precursor of miR-196a2 was shown to impact the risk of non-small cell lung cancer (Hu et al. 2008; Tian et al. 2009; Yuan et al. 2013), esophageal cancer (Ye et al. 2008), breast cancer (Hoffman et al. 2009; Hu et al. 2009), HCC, and liver cirrhosis (Li et al. 2010; Qi et al. 2010), as well as the overall survival of patients with advanced gastric cancer (Stenholm et al. 2013). This effect was explained by the fact that that presence of the rare T allele severely lowers synthesis of both miRs expressed from the precursor and leads to altered gene recognition by the variant miR-196a2-3p (Hoffman et al. 2009; Hu et al. 2008). Since the target genes for miR-196a include mediators of apoptosis and Hox genes, its aberrant expression can lead to severe changes in cellular pathways and initiate the process of tumorigenesis (Hornstein et al. 2005; Luthra et al. 2008).

Another polymorphism impairing the maturation of a miRNA and leading to inhibition of its function was rs895819 (A → G) within miR-27a. The SNP has been proved to modify the risk for breast cancer in some populations (Kontorovich et al. 2010; Yang et al. 2010), but the effect was not observed in others (Catucci et al. 2012). The overall protective role associated with the rs895819 heterozygous state was proven for Caucasians in a recent meta-analysis (Wang et al. 2013). However, in advanced gastric cancer the SNP was associated with worse overall survival (Stenholm et al. 2013).

Although information on germline polymorphisms in microRNAs is growing, the occurrence of somatic mutations is generally much less well understood and less commonly identified. Research in this area was for a long time not a focus of interest and data are still lacking. It seems, however, that information on miRNA-related somatic mutations will soon be expanded. A recently published database, SomamiR, lists 26 somatic mutations that potentially affect the functioning of microRNAs in various diseases (Bhattacharya et al. 2013).

### SNPs in microRNA target sites

Polymorphism in a 3′UTR of a gene may create as well as destroy a miRNA-binding site and thus play a role similar to a SNP located within a miRNA seed region. The importance of the miRNA:mRNA interactions for the proper functioning of a cell was highlighted by a study demonstrating a significantly lower frequency of SNPs in the miRNA-binding sites than in the entire 3′UTRs of genes (density 0.182 vs. 0.213 SNP/kilobase). These data indicate that such SNPs are presumably negatively selected under evolutional pressure (Yu et al. 2007). In consequence, the study underlined the role of miRNA disturbances in the pathogenesis of human diseases and, indeed, identified a number of SNPs with an aberrant allele frequency in human cancers. A recent release of miRdSNP, a database of disease-associated SNPs and microRNA-target sites located in 3′UTRs of human genes, includes 630 unique dSNPs and 786 SNP-disease associations (Bruno et al. 2012).

Studies have revealed the role of polymorphisms in microRNA recognition sites in the predisposition to particular malignancies, and by these means provided an additional proof of the implication of miRNA aberrations in the pathogenesis of cancers. A good example of a functional SNP located in a binding site for miRNA is rs61764370 (G → T) within the 3′UTR of *KRAS*. Presence of the SNP interferes with let-7 binding, weakens the inhibition of KRAS expression and leads to over-activation of the Raf, PI3K, and growth factor signaling pathways. Rs61764370 was associated with an increased risk for non-small cell lung cancer (Chin et al. 2008), and reduced survival in patients with oral cancer (Christensen et al. 2009). The rare rs61764370 variant was also shown to modulate the response to cetuximab, an epidermal growth factor receptor inhibitor, in metastatic colorectal cancer patients (Zhang et al. 2011).

Another miRNA-related SNP that was shown to alter the response to therapies was the 829C → T SNP in *DHFR* gene, located within the binding site for miR-24. Dihydrofolate reductase is a target of methotrexate, an important antimetabolite and antifolate agent used in the treatment of malignancies including acute lymphocytic leukemia, non-Hodgkin’s lymphoma, osteosarcoma, or choriocarcinoma. Overexpression of *DHFR* caused by the loss of miR-24 binding results in resistance to methotrexate (Mishra et al. 2007).

### MicroRNA length heterogeneity

**Fig. 1:**
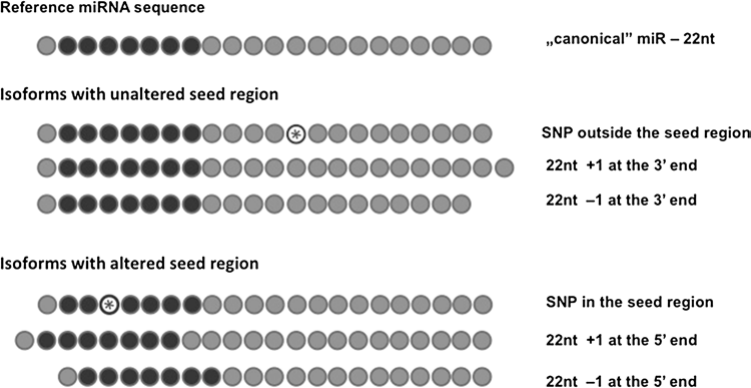
SNPs and length variations of microRNAs can alter the processing of a mature miR or directly change the seed region resulting in isoforms that regulate distinct target genes when compared to their canonical counterparts

Another intriguing subject related to the changes in miR sequences and the resulting alterations in their functioning is microRNA length heterogeneity. When next-generation sequencing was applied to microRNA analysis it became clear that an individual miRNA gene may give rise not only to the 5p and 3p mature miRNAs, but also to several additional miRNAs of varying length, named isomiRs (Kuchenbauer et al. 2008; Landgraf et al. 2007). Importantly, sequence variations of many of the isomiRs consist of the addition or deletion of nucleotides at their 5′end when compared to the reference miRNA deposited in miRbase. This results in a change of the miR’s seed region and, in consequence, leads to recognition and regulation of distinct sets of target genes. Thus, the effect of microRNA length variation can be similar to the effect mediated by microRNA polymorphisms (Fig. 1).

**Fig. 2:**
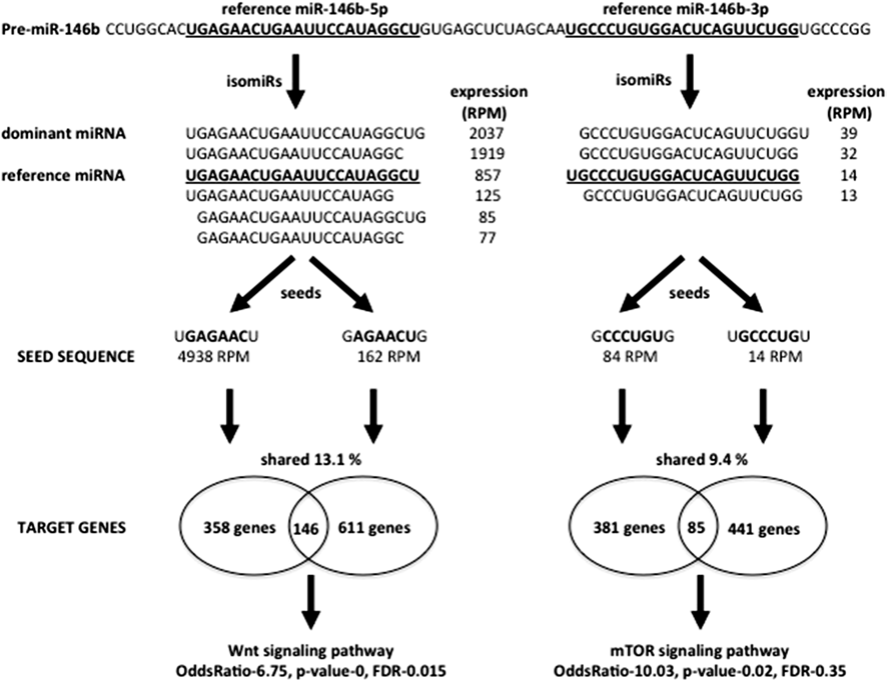
Functional pathways of pre-miR-146b. Pre-miR-146b produces several isoforms of two canonical miRNAs (miR-146b-5p and miR-146b-3p). The isomiRs have four alternative seeds sequences, potentially binding unique sets of target genes. Shared target genes cooperatively regulate two important pathways implicated in tumorigenesis—Wnt and mTOR. RPM-reads per million in papillary thyroid carcinoma samples (mean count) (modified from Swierniak et al. 2013)

The “templated” isomiRs originate from imperfect specificity of both Drosha and Dicer cleavage of microRNA precursors, mainly due to asymmetrical structural motifs present in precursor hairpins (Neilsen et al. 2012; Starega-Roslan et al. 2011). MicroRNAs can also be trimmed by exonucleases, or extended, mainly at their 3′ends, through the addition of ribonucleotides catalyzed by nucleotidyl transferases (Chiang et al. 2010; Cloonan et al. 2012; Kim 2005; Kuchenbauer et al. 2008; Landgraf et al. 2007; Lee et al. 2010; Swierniak et al. 2013). IsomiRs are tissue-specific and functionally active cooperative partners of canonical miRNAs (Cloonan et al. 2012). Although the role of isomiRs in the pathogenesis of cancers requires more studies, deregulation of isomiRs was already shown in a murine model of leukemia (Kuchenbauer et al. 2008) as well as in melanoma (Kozubek et al. 2013) and papillary thyroid carcinoma (Swierniak et al. 2013). Interestingly, all these studies showed that the most abundant mature sequence of most miRNAs differs from the canonical, reference sequence deposited in miRBase. In bone marrow cell lines, 336 identified miRNAs were expressed in 3,390 isoforms and the number of different isomiRs for a given miRNA ranged from 1 to 74 (Kuchenbauer et al. 2008). Another study identified isomiRs that exhibited different expression levels between primary cutaneous melanoma and metastatic melanoma (Kozubek et al. 2013). A recent study identified 427 reference microRNAs expressed in the thyroid gland, while the number of significantly expressed isomiRs reached 1749 (Swierniak et al. 2013). Many of the identified isomiRs were deregulated in cancer when compared to control tissue. One of the most striking examples of deregulated miRs were the products of the mir-146b precursor. Mir-146b is processed to two mature microRNAs: miR-146b-3p and miR-146b-5p. In thyroid tissue, these microRNAs are expressed altogether in 10 isoforms, and their expression in tumor samples was 14.4–39 times higher than in control tissue. In both cases, the reference sequences were not the dominant ones (Swierniak et al. 2013). The isoforms of both mature miRs vary at their 5′ends with a 1nt-difference, creating 2 alternative seeds each. In silico target prediction revealed that each seed binds a unique set of target genes, and only 13.1 and 9.4 % of the target genes are regulated by the two highly expressed seeds of miR-146b-5p and miR-146b-3p, respectively (Fig. 2). The target genes shared by different isomiRs seem to be of particular interest, because their regulation is independent from the biases of Dicer activity. Moreover, the high number of isoforms increases their regulatory effect. The analysis of target genes concertedly regulated by both alternative seeds of miR-146b-5p and miR-146b-3p showed an overrepresentation of two pathways involved in tumorigenesis, namely Wnt-signaling pathway (Odds ratio 6.75, FDR 0.015), and mTOR-signaling pathway (Odds ratio 10.03, FDR 0.35), respectively (Swierniak et al. 2013, and unpublished data, Fig. 2).

Although microRNA research is a relatively young subject, a significant number of studies show the importance of microRNA-related sequence variations in the pathogenesis and susceptibility to particular malignancies. It is worth mentioning that the coordinate action of microRNA expression and sequence variations can differentially predispose to diseases in different populations, and can play divergent roles in different tissue types. As the number of identified miRs and polymorphisms are growing, this area will be constantly expanded and bring new insight into the role of miRs in human cancers.
